# A diverse array of genetic factors contribute to the pathogenesis of Systemic Lupus Erythematosus

**DOI:** 10.1186/1750-1172-8-2

**Published:** 2013-01-07

**Authors:** Nicki Tiffin, Adebowale Adeyemo, Ikechi Okpechi

**Affiliations:** 1 South African National Bioinformatics Institute/MRC Unit for Bioinformatics Capacity Development, University of the Western Cape, Private Bag X17, Bellville, Cape Town, 7535, South Africa; 2 Centre for Research on Genomics and Global Health, National Human Genome Research Institute, Bethesda, MD, USA; 3 Division of Nephrology and Hypertension, Department of Medicine, University of Cape Town, Cape Town, South Africa

**Keywords:** Systemic lupus erythematosus, Autoimmunity, Genetic susceptibility, Apoptosis, dsDNA, Disease genes

## Abstract

Systemic lupus erythematosus (SLE) is a chronic systemic autoimmune disease with variable clinical presentation frequently affecting the skin, joints, haemopoietic system, kidneys, lungs and central nervous system. It can be life threatening when major organs are involved. The full pathological and genetic mechanisms of this complex disease are yet to be elucidated; although roles have been described for environmental triggers such as sunlight, drugs and chemicals, and infectious agents. Cellular processes such as inefficient clearing of apoptotic DNA fragments and generation of autoantibodies have been implicated in disease progression. A diverse array of disease-associated genes and microRNA regulatory molecules that are dysregulated through polymorphism and copy number variation have also been identified; and an effect of ethnicity on susceptibility has been described.

## Introduction

**Figure 1:**
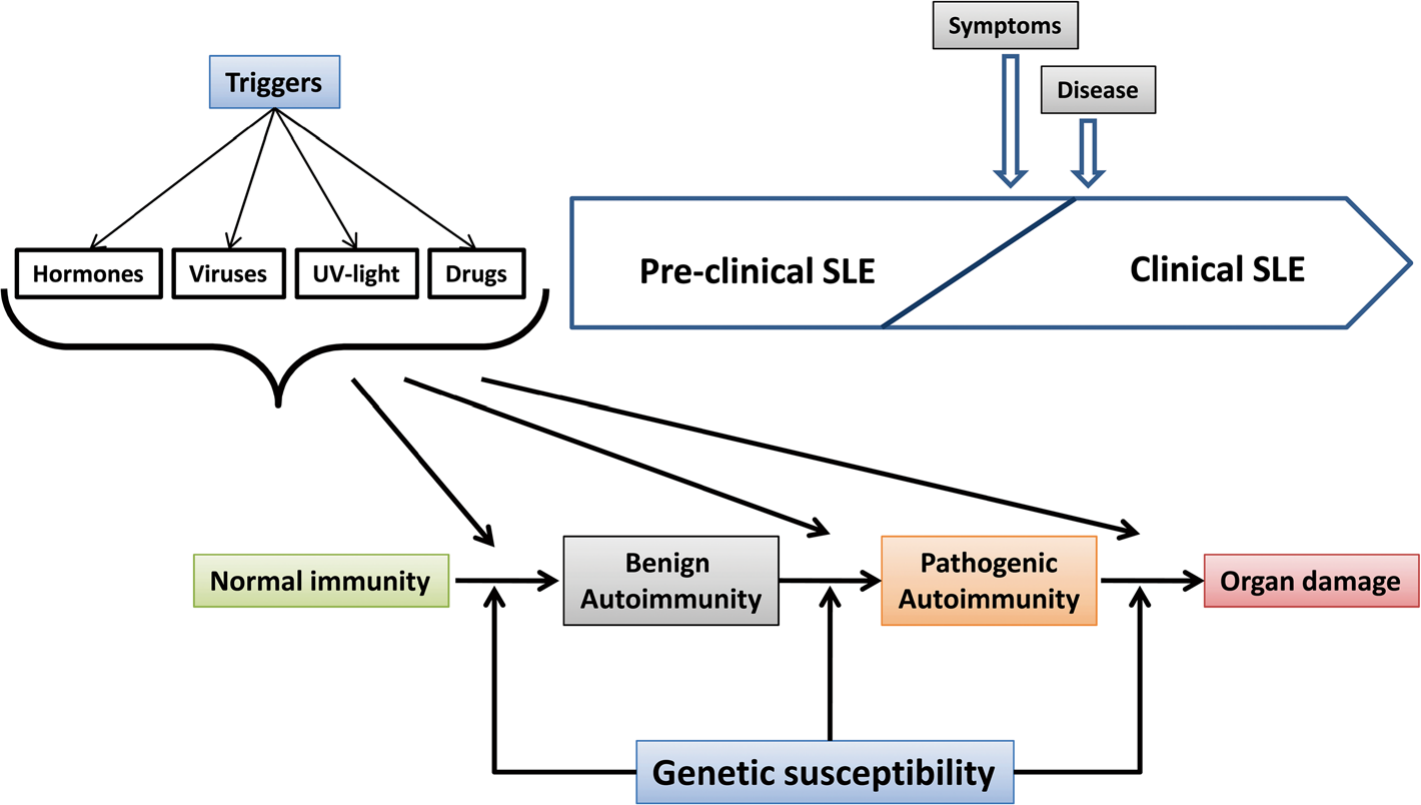
**Stages in the pathogenesis of SLE.** Environmental triggers (hormones, viruses etc.) and genetic factors along with other chance events, act on the immune system to initiate autoimmunity. Symptoms of clinical illness appear soon after pathogenic autoimmunity develops.

Systemic lupus erythematosus (SLE, “disseminated lupus erythematosus”, ORPHA536) is a chronic systemic autoimmune disease with variable clinical presentation. SLE commonly affects the skin, joints, haemopoietic system, kidneys, lungs and central nervous system, although all organs can be implicated and the involvement of major organs can be life-threatening. The exact pathological mechanisms of SLE remain elusive, and the aetiology of SLE is known to be multifactorial, involving multiple genes, sex hormones, and environmental factors including sunlight, drugs and infections (especially Epstein-Barr virus, EBV) [1] (Figure 1). With the appropriate genetic background, presence of immune triggers, and effective immune system activation, SLE can manifest - although disease-specific antibodies may circulate for up to five years before the first clinical signs of organ involvement in the disease [2,3].

The development of SLE can be categorized into several phases with a cumulative effect. Initially, an interplay between environment, hormonal and genetic factors results in decreased immunologic tolerance towards certain self antigen. This systemic autoimmunity then results in increased serum antinuclear and anti-glomerular autoantibodies, leading to an enhanced autoimmune repertoire; and aberrations in both the innate and adaptive arms of the immune system play an important role in the genesis and progression of lupus. Finally, immunological events occur within the target organ and result in end organ damage [4,5].

Studies of racial tendencies show that SLE occurs more frequently in non-Caucasian individuals, supporting a role for genetic predisposition to SLE. In America, SLE is more frequent in African-Americans, Hispanics and Asians than in Caucasians, and has been described to be three to four times higher among African-American women compared to Caucasian women [6]. The past half century has seen a ten-fold increase in the annual incidence of SLE in industrialized Western countries [7,8], with estimates of prevalence in the UK at 25 per 100 000, and incidence approximately 1 (males) - 8 (females) per 100 000 [9-11]. The epidemiology of SLE in the developing world remains largely unknown due to poor disease recognition, poor diagnostic tools and supposed “rarity” of SLE in tropical areas [12-14]; people of African and Asian extraction living in industrialized countries, however, demonstrate the highest prevalence rates in the world [7,15]. Racial admixture [16] and increased exposure to environmental factors such as tobacco products and viral infections are thought to increase the risk in people of African or Asian extraction living in industrialized countries [17]. Tropical infections such as malaria, on the other hand, appear to offer protection from SLE [18]. Although some of these differences in population prevalence of SLE may be explained by the effects of environmental differences, genetic differences between populations clearly contribute to the complexities of SLE pathogenesis [19].

### Cellular mechanisms underlying SLE

**Figure 2:**
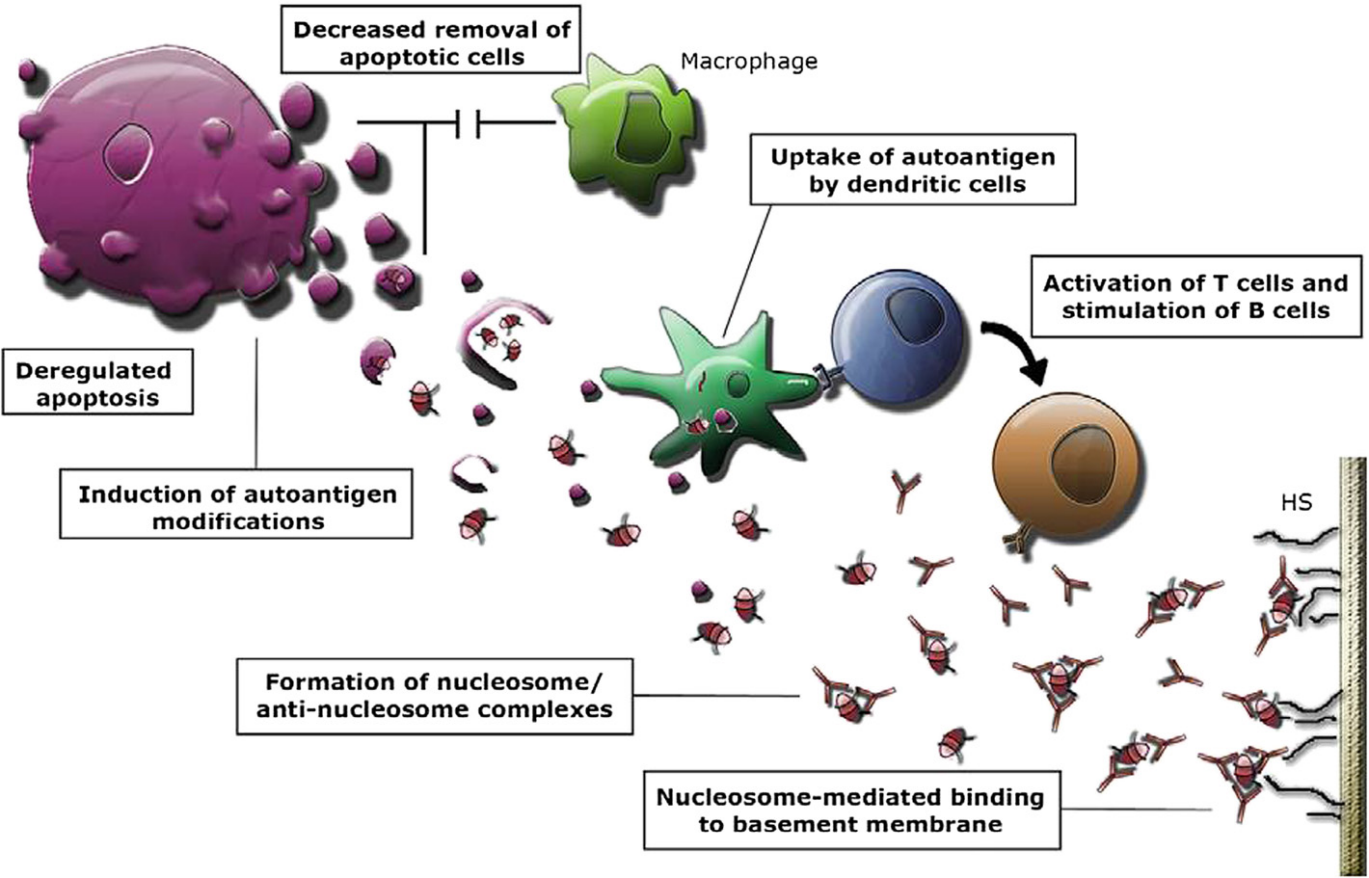
**General hypothesis for the pathogenesis of SLE.** Increased production of apoptotic blebs and/or reduced clearance of apoptotic blebs lead to the release of chromatin into the circulation. Presence of chromatin in circulation leads to the activation of antigen-presenting cells (APCs) and the formation of pathogenic immune complexes that incite glomerulonephritis. From Munoz et al. 2008 [20], copyright © 2011 by SAGE Publishing, reprinted by permission of SAGE.

A core hypothesis for SLE pathogenesis implicates poorly cleared or excessively produced apoptotic blebs as a constant source of partially degraded nucleosomes (Figure 2) [20]. Impaired clearance of dying cells in SLE may explain the accumulation of apoptotic cells in tissues, while secondary necrosis of these cells might contribute to the chronic inflammation that is seen in this disease. Abnormal phagocytosis of apoptotic cells in clinical and experimental studies of lupus has been demonstrated [21,22]; and furthermore, Bijl et al. have demonstrated abnormal phagocytosis in SLE patients, showing reduced uptake of apoptotic cells by monocyte-derived macrophages as a serum-dependent defect that is associated with decreased levels of C1q, C4, and C3 [22].

During apoptosis, several proteins and nuclear materials (DNA and RNA) are modified by cleavage, facilitating specific, apoptosis-induced post-translational modifications of autoantigens (e.g. methylation, phosphorylation, ubiquitination and citrullination). Normally apoptotic cells are quickly removed by phagocytosis before release of their modified contents. In SLE, however, removal of apoptotic cells is dysregulated and the blebs and their modified contents are exposed to the immune system at the cell surface, resulting in recognition as non-self antigens (danger signaling) [23]. Dendritic cells (DCs) become activated by the modified autoantigens, leading to an immunogenic response and the formation of autoantibodies. Autoimmunity in SLE thus occurs when self-molecules evoke an immunologic challenge that activates the immune system and stimulates host defense mechanisms [24]. This ‘danger’ signaling can induce autoimmunity in susceptible individuals through exogenous (pathogen-associated molecular patterns – PAMPs) or endogenous (damage-associated molecular patterns – DAMPs) pathways, in which DNA and RNA exhibit important immunological activity [25]. The PAMPs can also activate effector and regulatory T-cells, break tolerance and stimulate self-reactive B-cells [26]. Recent evidence has shown that toll-like receptors (TLRs) associated with these danger molecules mediate the signaling pathways that over-ride the peripheral tolerance mechanisms, and promote and sustain chronic inflammation and autoimmune diseases [27,28]. Engagement of TLRs may serve two functions: (i) up-regulation of co-stimulatory molecules (CD80/CD86) which play an important role in the activation of chromatin-specific T helper cells [29], and (ii) up-regulation of certain matrix metalloproteases (MMP2 and MMP9) with the potential to cause significant damage in the kidney in SLE patients [30,31]. Taken together, apoptosis and poor clearance of apoptotic materials are therefore key processes in the pathogenesis of SLE.

### Genetic factors contributing to SLE

High heritability, monozygotic and dizygotic twin studies [32-34], and incidence in first and second-degree relatives [35,36] and siblings [37] all indicate a substantial genetic component to SLE. Many linkage and association studies also indicate regions of the genome associated with the disease. Finally, a clear demonstration of susceptibility differences by different ethnic groups suggest that genetic diversity underlies such differences, and that certain genetic backgrounds may alter the likelihood of developing SLE (reviewed in [38]).

Aetiological genes for SLE were initially identified by a hypothesis-driven approach, where candidate genes were assayed for variants prevalent in patients compared to healthy controls. The candidate gene approach best identifies single genes of high aetiological effect, in a Mendelian model of disease where mutation of one gene causes a disease phenotype. Disease gene identification has since shifted to a complex model of disease genetics, in which multiple genes have small effects that together contribute to the disease phenotype [39]. New technologies enable genome-wide association studies, where disease association with all SNPs across the genome can be tested in one experiment, [40-42] requiring no pre-existing hypotheses about the disease mechanisms, and generating new hypotheses about disease mechanisms. Furthermore, next generation sequencing techniques make more attainable the sequencing of entire genomes of patients and case controls in order to identify aetiological variants [43]. Candidate gene studies have identified multiple aetiological variants in MHC class II receptor, Fcγ receptor gene and complement cascade (C1a, C2 or C4) gene families.

Linkage analyses of affected families have identified SLE-susceptibility loci containing strong candidate genes, but in general have not provided necessary resolution to identify individual disease variants. Genome-wide association studies (GWAS), however, have rapidly increased the identification of SLE genes. The majority of GWAS for SLE to date are on European and Asian population patient/control populations (reviewed in [38], [44]), with limited studies being conducted in African American populations despite a higher incidence of SLE in this group [45]. Genes that have been identified to date as causative genes for SLE are summarised in Additional file 1: Table S1.

#### Functions of SLE-associated genes

The genes that have been identified to date as aetiological genes for SLE are predominantly implicated in immunity and immunoglobulin binding, and inflammatory response. Analysis of Gene Ontology functional annotation of these genes, as described in [46], shows the top five most significantly overrepresented functions to be “protein binding”, “immune system process”, “immune response”, “immunoglobulin binding” and “protein complex binding”. The top thirty associated Gene Ontology annotations are shown in Additional file 2: Table S2 and Additional file 3: Diagram S3. These terms are consistent with a phenotype that entails activation of an autoimmune response, resulting in aggregation of immune complexes.

**Figure 3:**
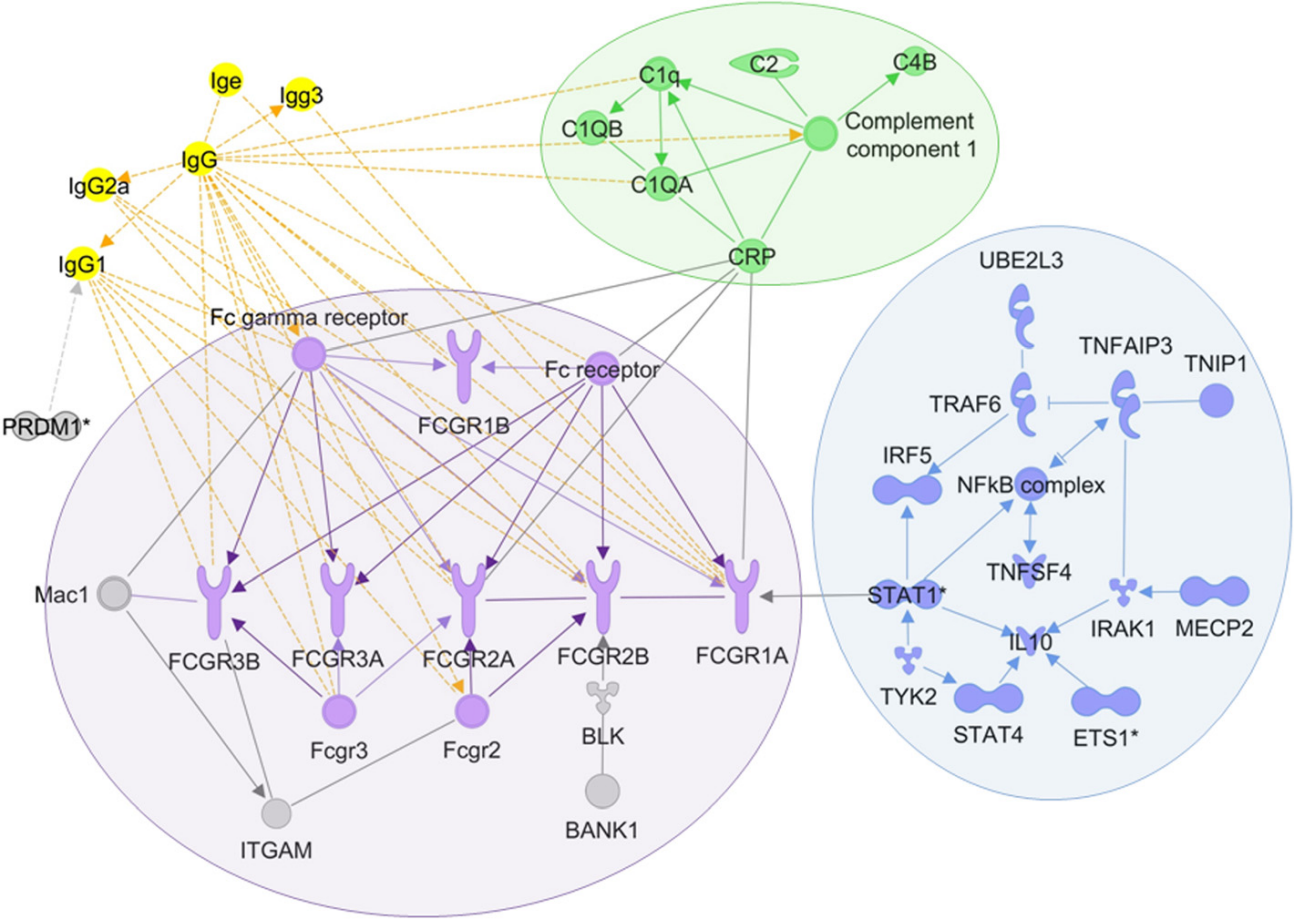
**Network analysis of known SLE**-**associated genes.** Network analysis using Ingenuity Pathway Analysis software shows regulatory interactions between almost half of the known SLE-associated genes, shown as shaded molecules. Three sub-networks are circled. Potential regulatory partners that participate in the networks but are not previously associated with SLE are shown as non-shaded molecules. Several IgG molecules have multiple interactions with network members, shown as dotted lines.

Ingenuity pathway analysis (Ingenuity Systems, http://www.ingenuity.com) of SLE-associated genes shows enrichment of representation in well-defined canonical pathways. The top five enriched pathways are “dendritic cell maturation” (p = 7.3 × 10^-13^), “IL-10 signaling” (p = 2.09 × 10^-6^), “complement system” (p = 2.81 × 10^-6^), “systemic lupus erythematosus signaling” (p = 3.81 × 10^-6^), and iNOS signaling (p = 1.05 × 10^-5^). Of interest, an initial regulatory network analysis shows a high degree of interaction between 33 of the 67 molecules analysed, with three clear sub-networks appearing (Figure 3): complement-related molecules form one sub-network, and are connected to the second sub-network containing Fc-gamma receptors and their interacting molecules through the binding of CRP to Fc receptor molecules. A third sub-network implicates the NFkappaB (NF-kB) complex and IL-10 in the interactions between signal transduction molecules (STATs, TYK2, IRAK1) and their binding and regulatory partners; and this sub-network connects to the Fc receptor network through the interaction of STAT1 with FCGR1A. Many molecules in the complement and Fc gamma receptor sub-networks also interact with Ig G molecules. The top five upstream transcription factors most commonly regulating the known SLE-associated genes are the NF-kB complex - and more specifically NFKB1, HDAC11, ZNF148 and STAT6. NF-kB has been implicated in inflammatory disease [47]; inhibition of the HDAC family has been previously demonstrated to play a role in models of lupus ([48], reviewed in [49]); and a role for STAT6 in lupus has been postulated in an association study in Chinese patients [50].

Although a substantial list of genes is associated with SLE through GWAS and candidate gene studies, it is still unclear how these genes may be contributing to the disease phenotype, and this is also confounded by the complex disease model where multiple genes are anticipated to each make small contributions to the disease state [51]. Also, in many cases the genes are associated to SLE through the ‘tagging SNPs’ – so the identified SNP is not necessarily aetiological but rather a marker for the discrete region of the genome (haploblock) containing the disease variant [52].

#### SLE- associated single nucleotide polymorphisms

Altering a single base within the gene sequence can cause an altered, or disease phenotype. Changing the DNA sequence can result in a different amino acid appearing in the translated protein (a non-synonymous SNP); or a deletion or insertion of bases can cause a frameshift mutation in the DNA whereby the protein structure is significantly altered and often prematurely truncated. SNPs in the regulatory sequence around a gene may result in changes in the rate of synthesis or degradation of proteins, or alter mRNA splicing events that define final gene structure. To date, no single SNP has been associated with a functional change at the protein level in patients with SLE (although a recent study describes reduced Ets1 binding to the promoter of miR-146a due to a functional SNP, resulting in reduced expression, described in section 7.2.4 [53]). With increased understanding of genetics underlying disease, however, new elements of gene regulation are being investigated in the disease state, yielding some surprising results in SLE cohorts.

#### Gene copy number variation in SLE

Copy number variation (CNV) arises when a section of the genome containing an entire gene or genes is replicated or deleted, causing extra copies of the gene in one individual compared to another [54]. The net effect of CNV is commonly an alteration in gene expression – altered gene ‘dosage’ - with a consequent downstream amplification or attenuation of the gene’s function. CNV has been shown for SLE-associated genes: low copy number of complement component 4 (C4a/C4b) [55] and Fcγ receptor 3B (FCGR3B) genes increases risk of SLE, whereas more copies of these genes have a protective effect. Complement component 4 is involved in the clearance of apoptotic debris and immune complexes [56], and an increase in complement expression could increase this function and thus protect against the aggregation of antibodies seen in SLE. Additionally, deficiency of C4 has been extensively reported in SLE patients (reviewed in [57]). The Fcγ receptors bind the Fc domain of IgG antibodies and regulate immune responses via tyrosine phosphorylation of their active cytoplasmic domains. FCGR3B is a functional regulator of neutrophil activation through altered IgG binding, and had been known to play a role in susceptibility to, and severity of SLE (reviewed in [57]).

#### Dysregulation of microRNA in SLE

MicroRNAs (miRNA) are regulatory molecules that are increasingly implicated in transcriptional dysregulation associated with disease [58,59]. These are short (25 nucleotide) single-stranded non-coding RNA molecules that are processed from primary transcripts into stem-loop-stem structures and finally to functional single stranded RNA. This processed miRNA is complementary to a section of the target mRNA molecule, and will thus bind to and inhibit mRNA translation or initiate mRNA degradation (reviewed in [60]). miRNA molecules regulate transcriptional networks in this way, with central roles in some cancers, cell development, inflammatory response and neurodegenerative disorders [61-63]. The role of miRNA molecules in regulation of innate and adaptive immunity and autoimmunity has been reviewed extensively; and pertinent to the predominance of SLE (and other autoimmune diseases) in women, the regulation of immune system miRNAs by estrogen is also discussed [59,64].

In 2007, Dai et al. [65] examined miRNA expression in peripheral blood mononuclear cells (PBMC) from 23 SLE patients compared to 10 healthy controls, indentifying seven consistently downregulated miRNAs in the disease state (miR-196a, miR-17-5p, miR-409-3p, miR-141, miR-383, miR-112 and miR-184), and nine upregulated miRNAs (miR-189, miR-61, miR-78, miR-21, miR-142-3p, miR-342, miR-299-3p, miR-198 and miR-298). In further studies on a subset of SLE patients, 36 upregulated and 30 downregulated miRNAs were identified in lupus nephritis (LN) patients compared to controls [66]; and 29 and 50 differentially expressed miRNAs were found in African American and European American LN patients respectively [67]. Further studies identified MiR-148a and MiR-21 as key microRNA molecules in lupus, with a role for both in DNA hypomethylation in the disease state [68]. MiR-21 is again implicated in SLE, with a proposed role in T-cell response through regulation of PDCD4 [69]. MiRNA-126 contributes to SLE by targeting DNA methylation [70], and downregulation of miR-181-a has been associated with paediatric cases of SLE [71]. An assay of miRNA-146a in PBMCs shows downregulation in SLE patients in two independent studies [72,73], and underexpression of this microRNA may underlie SLE through dysregulation of the type 1 interferon pathway [73]. Recently, a SNP in the promoter of miR-146a was shown to decrease binding of the transcriptional factor Ets1 with concomitant decreased expression of the microRNA molecule. This may in turn cause upregulation of the type I IFN pathway, as seen in these patients [53]. Decreased levels of miR-146a (and miR-155) in serum from SLE patients has been shown in a further study [74], and the level of miR-155 is shown to be downregulated in regulatory T-cells from SLE patients [75].

The type of microRNA dysregulation associated with SLE can also be indirect, for example Divekar et al. [75] also show downregulation of gene expression for Dicer in regulatory T-cells from SLE patients. Dicer is the endoribonuclease that processes precursor microRNA molecules to generate functional microRNAs (described in [76]), suggesting that the milieu of active microRNA molecules may generally be altered in regulatory T-cells from SLE patients due to changes in miRNA processing. In another study, Hikami et al. [77] show that in a cohort of SLE patients, a disease-associated polymorphism in the 3’-untranslated region of the *SPI1* gene falls in a binding region for miR-569.

There is ever-growing evidence that microRNA regulation is altered in the disease state; and specifically in SLE. Further research in this field will need to bring together the different strands of evidence for a more cohesive picture of microRNA regulation, and dysregulation in SLE. A summary of some of the miRNA molecules implicated in SLE is shown in Additional file 4: Table S4.

#### Mouse models for SLE

Several mouse strains spontaneously develop a disease that closely resembles SLE, resulting in the production of autoantibodies, followed by development of immune molecule complexes in the kidneys with associated damage; and include the strains MRL-*Fas*^*lpr*^, BXSB.*Yaa,* the F_1_ hybrid between NZB and NZW, and inbred derivatives of these strains [78]. Over 100 regions in the mouse genome have been associated with SLE in the mouse by linkage analysis. These regions are called quantitative trait loci (QTL) and are extensively reviewed by Morel (2010) [79]. Some mouse SLE QTL can also be shown to overlap with human QTL associated with lupus heritability [80]. With extensive progress in generating knock-out mouse models and using new technologies to define existing mouse models, the number of mouse models available to study SLE is on the increase: a comprehensive list of 45 mouse models currently associated with the disease SLE can be obtained by a simple search of the Mouse Genome Informatics database, a database hosted by the Jackson Laboratory, USA (http://www.informatics.jax.org/) [81]. The orthologous disease-associated genes for both mouse and human are also clearly documented and show the extent of the overlap between disease genes for the two species. The results obtained by this search are shown in Additional file 5: Table S5. Availability of information on human genetics underlying SLE has made it increasingly possible to verify that the functional pathways underlying pathogenesis in the two species are similar [79,82]. Thus parallel research into disease genetics underlying mouse models of SLE can inform research into the human disease, and similarly progress made in understanding genetics underlying the human disease can refine mouse models further. An example of this is the use of mouse models of SLE in the investigation of microRNA expression patterns in SLE [83].

### Summary

Research to date has identified multiple facets of SLE, including a better understanding of the cellular and environmental processes leading to the disease state as well as genetic abnormalities that are associated with the disease. There have been many advances in understanding genetic factors that are associated with the disease – in many cases through GWAS – but there is still a pressing need to interpret such factors with regard to their biological impact. The way in which fundamental immune and biological responses are perturbed by these genetic factors needs to be better understood before there can be similar advances in the diagnostic, prognostic and therapeutic management of SLE for maximum benefit to the patient.

## Abbreviations

SLE: Systemic lupus erythematosus; RNA: Ribonucleic acid; DNA: Deoxyribonucleic acid; PAMPs: Pathogen-associated molecular patterns; DAMPs: Damage-associated molecular patterns; TLRs: Toll-like receptors; EBV: Epstein-Barr virus; LN: Lupus nephritis; GWAS: Genome-wide association study; SNP: Single Nucleotide Polymorphism; CNV: Copy number variation; miRNA: Micro-ribonucleic acid; QTL: Quantitative trait loci.

## Competing interests

The authors have no competing interests.

## Authors’ contributions

NT, AA and IO drafted and wrote the manuscript. All authors read and approved the final manuscript.

## Supplementary Material

Additional file 1 **Table S1.** Genes associated with SLE. Click here for file

Additional file 2 **Table S2.** Top 30 gene ontology functional annotations for SLE candidate genes [84,85]. Click here for file

Additional file 3 **Diagram S3.** Relationships between top gene ontology functional terms for SLE candidate genes. Click here for file

Additional file 4 **Table S4.** microRNA molecules implicated in SLE [86]. Click here for file

Additional file 5 **Table S5.** Mouse models for SLE. Click here for file
