# Supplementary material for: A diverse array of genetic factors contribute to the pathogenesis of Systemic Lupus Erythematosus
Source: Orphanet J Rare Dis. 2013 Jan 7;8:2. doi: 10.1186/1750-1172-8-2 (PMC3551738; doi:10.1186/1750-1172-8-2)
Supplement: Additional file 1 — Table S1. Genes associated with SLE. [file 1750-1172-8-2-S1.pdf]

Supplementary data table S-1: Genes Implicated in Systemic Lupus Erythematosus.

Symbol	Chromosome	SLE	LN	Caucasians	African American	Asian	Hispanic	CNV	epigenetics	miRNA
ATG5	6	YES [1]′[2]		YES [1]′[3]		YES [4]				
ACTN4	19	YES [5]		YES [5]						
BANK1	4	YES [6]		YES [6]						
BLK	8	YES [1]′[7]′[8]		YES [7]′ [1]′[8]′[3]		YES [4]′[9]				
C1QA	1	YES [10]′[11]′[12]	YES [13]							
C1QB	1	YES [10]′[11]′[12]	YES [13]							
C2	6	YES [10]′[11]′[12]								
C4A	6	YES [10]′[11]′[12]′ [14]		YES [14]				YES [14] (caucasians)		
C4B	6	YES [10]′[11]′[12]′ [14]		YES [14]				YES [14] (caucasians)		
ATF6B (CREBL1)	6	YES [15]								
CRP	1	YES [16]		YES [17]′[18]′[19]	YES [17]′[18]′[19]					
CD44	11	YES [5]		YES [5]						
DNAJA1	9	YES [5]		YES [5]						

ETS1	11					YES [4]′[9]				
FCGR1A	1	YES [16]								
FCGR1B	1	YES [16]								
FCGR2A	1	YES [16]	YES [20]	YES [1]						
FCGR2B	1	YES [16]								
FCGR3A	1	YES [21]	YES [22]′[20]		YES [23]		YES LN [22]			
FCGR3B	1	YES [21]′[24]	YES glomerulo-nephritis [25]			YES [22]′[26]		YES [25]′ [27]′[28]		
FRK-PTPRD pair	6; 9	YES [5]		YES [5]						
HLA-DRB1		YES [15]		YES [3]′[15]		YES [4]′ [9]				
HLA-DRB2	6	YES [29]	NO [30]′[31]	YES [29]	NO [29]	YES [4]′[9]				
HLA-DRB3	6	YES [29]	NO [30]′[31]	YES [29]′ [7]	NO [29]	YES [4]′[9]				
ICA1	7	[1]								
IKZF1	7			YES [3]		YES [4]′				
IL10	1			YES [1]′ [3]						
IL21	4	YES [32]		YES [32]	YES [32]					
IRAK1 (MECP2)	X	YES [33]		YES [3]						YES mir146 [34]
IRF5	7	YES [35, 36]		YES [1]′ [3]		YES [4]′[9]				YES mir146 [34]

IRF53 (not determined)				YES [7]′ [8]						
IRF8	16	YES [5]		YES [5]						
ITGAM	16	YES [1]′[7]′[37]		YES [38]′ [7]′ [1]′ [8]′[3]	YES [38]	NO [38]				
JAZF1	7			YES [1]′[3]						
KPNA1	3	YES [5]		YES [5]						
LRRC18 (WDFY4)						YES [4]′[9]				
Lyn				YES [1]						
MECP2	X	YES [39]′[40]′[41]								
MICB	6	YES [15]		YES [15]						
NMNAT2	1	YES [1]								
NOTCH4	6	YES [42]								
OR2H2	6	YES [15]		YES [15]						
PDCD1	2	YES [43]	YES [44]′[45]′[46]′ [47]							

PHRF1	11			YES [1]′[3]						
PPP2CA	5	YES [48]			NO [48]	YES [48]	YES [48]			
PRDM1 (ATG5)	6			YES [1]′[3]						
PTN	7	YES [5]		YES [5]						
PTPN22	1	YES [49]		NO [50]′ YES [1]′ [3]						
PTTG1	5	YES [1]′[2]								
PXK	3			YES [1]′[1]′[3]						
RABGAPIL	1	YES [5]		YES [5]						
RASGRP3	2					YES [4]				
RPS6KA1	1	YES [5]		YES [5]						
SERPIND1-PARVB pair	22′ 22	YES [5]		YES [5]						
SCUBE1				YES [1]						
SKIV2L	6	YES [51]								
SLC15A4	12			YES [3]		YES [4]				
SPP1	4	YES [52]								
STAT1	2									YES mir146 [34]

STAT4	2	YES [53]	YES [54]	YES [7]′ YES [1]′ [8]′ [3]		YES [4]′ [9]				
SYK	9	YES [5]		YES [5]						
TAP2	6	YES [55]								
TNFAIP3	6	YES [8]		YES [8]′[3]		YES [4]′ [9]				
TNFSF4	1	YES [56]′[57]		YES [3]		YES [4]′				
TNIP1	5			YES [1]′[3]		YES [4]′				
TRAF6	11									YES mir146 [34]
TREX1	3	YES [58]								
TRIM27	6	YES [15]		YES [15]						
TRIM31	6	YES [42]								
TYK2	19	YES [35]′[39]′[3]								
UBE2L3 (HIC)	22	YES [1]′[2]′[5]		YES [1]′ [3]		YES [4]				
UHRF1BP1	6			YES [1]′ [3]						
XKR6				YES [1]						

References

1. Harley JB, Alarcon-Riquelme ME, Criswell LA, et al. Genome-wide association scan in women with systemic lupus erythematosus identifies susceptibility variants in ITGAM, PXK, KIAA1542 and other loci. Nat Genet 2008;40(2):204-10.
2. Graham RR, Hom G, Ortmann W, Behrens TW. Review of recent genome-wide association scans in lupus. J Intern Med 2009;265(6):680-8.
3. Gateva V, Sandling JK, Hom G, et al. A large-scale replication study identifies TNIP1, PRDM1, JAZF1, UHRF1BP1 and IL10 as risk loci for systemic lupus erythematosus. Nat Genet 2009;41(11):1228-33.
4. Han JW, Zheng HF, Cui Y, et al. Genome-wide association study in a Chinese Han population identifies nine new susceptibility loci for systemic lupus erythematosus. Nat Genet 2009;41(11):1234-7.
5. Ramos PS, Williams AH, Ziegler JT, et al. Genetic analyses of interferon pathway-related genes reveals multiple new loci associated with systemic lupus erythematosus (SLE). Arthritis Rheum 2011.
6. Kozyrev SV, Abelson AK, Wojcik J, et al. Functional variants in the B-cell gene BANK1 are associated with systemic lupus erythematosus. Nat Genet 2008;40(2):211-6.
7. Hom G, Graham RR, Modrek B, et al. Association of systemic lupus erythematosus with C8orf13-BLK and ITGAM-ITGAX. N Engl J Med 2008;358(9):900-9.
8. Graham RR, Cotsapas C, Davies L, et al. Genetic variants near TNFAIP3 on 6q23 are associated with systemic lupus erythematosus. Nat Genet 2008;40(9):1059-61.
9. Yang W, Shen N, Ye DQ, et al. Genome-wide association study in Asian populations identifies variants in ETS1 and WDFY4 associated with systemic lupus erythematosus. PLoS Genet 2010;6(2):e1000841.
10. Agnello V, De Bracco MM, Kunkel HG. Hereditary C2 deficiency with some manifestations of systemic lupus erythematosus. J Immunol 1972;108(3):837-40.
11. Hauptmann G, Grosshans E, Heid E. Lupus erythematosus syndrome and complete deficiency of the fourth component of complement. Boll Ist Sieroter Milan 1974;53(1):suppl:228.
12. Nishino H, Shibuya K, Nishida Y, Mushimoto M. Lupus erythematosus-like syndrome with selective complete deficiency of C1q. Ann Intern Med 1981;95(3):322-4.
13. Pickering MC, Botto M, Taylor PR, Lachmann PJ, Walport MJ. Systemic lupus erythematosus, complement deficiency, and apoptosis. Adv Immunol 2000;76:227-324.

14. Yang Y, Chung EK, Wu YL, et al. Gene copy-number variation and associated polymorphisms of complement component C4 in human systemic lupus erythematosus (SLE): low copy number is a risk factor for and high copy number is a protective factor against SLE susceptibility in European Americans. Am J Hum Genet 2007;80(6):1037-54.
15. Barcellos LF, May SL, Ramsay PP, et al. High-density SNP screening of the major histocompatibility complex in systemic lupus erythematosus demonstrates strong evidence for independent susceptibility regions. PLoS Genet 2009;5(10):e1000696.
16. Brown EE, Edberg JC, Kimberly RP. Fc receptor genes and the systemic lupus erythematosus diathesis. Autoimmunity 2007;40(8):567-81.
17. Edberg JC, Wu J, Langefeld CD, et al. Genetic variation in the CRP promoter: association with systemic lupus erythematosus. Hum Mol Genet 2008;17(8):1147-55.
18. Jonsen A, Gunnarsson I, Gullstrand B, et al. Association between SLE nephritis and polymorphic variants of the CRP and FcgammaRIIIa genes. Rheumatology (Oxford) 2007;46(9):1417-21.
19. Russell AI, Cunninghame Graham DS, Shepherd C, et al. Polymorphism at the C-reactive protein locus influences gene expression and predisposes to systemic lupus erythematosus. Hum Mol Genet 2004;13(1):137-47.
20. Karassa FB, Trikalinos TA, Ioannidis JP. The Fc gamma RIIIA-F158 allele is a risk factor for the development of lupus nephritis: a meta-analysis. Kidney Int 2003;63(4):1475-82.
21. Wu J, Edberg JC, Redecha PB, et al. A novel polymorphism of FcgammaRIIIa (CD16) alters receptor function and predisposes to autoimmune disease. J Clin Invest 1997;100(5):1059-70.
22. Kyogoku C, Dijstelbloem HM, Tsuchiya N, et al. Fcgamma receptor gene polymorphisms in Japanese patients with systemic lupus erythematosus: contribution of FCGR2B to genetic susceptibility. Arthritis Rheum 2002;46(5):1242-54.
23. Salmon JE, Millard S, Schachter LA, et al. Fc gamma RIIA alleles are heritable risk factors for lupus nephritis in African Americans. J Clin Invest 1996;97(5):1348-54.
24. Ptacek T, Li X, Kelley JM, Edberg JC. Copy number variants in genetic susceptibility and severity of systemic lupus erythematosus. Cytogenet Genome Res 2008;123(1-4):142-7.
25. Aitman TJ, Dong R, Vyse TJ, et al. Copy number polymorphism in Fcgr3 predisposes to glomerulonephritis in rats and humans. Nature 2006;439(7078):851-5.
26. Lee YH, Ji JD, Song GG. Fcgamma receptor IIB and IIIB polymorphisms and susceptibility to systemic lupus erythematosus and lupus nephritis: a meta-analysis. Lupus 2009;18(8):727-34.
27. Fanciulli M, Norsworthy PJ, Petretto E, et al. FCGR3B copy number variation is associated with susceptibility to systemic, but not organ-specific, autoimmunity. Nat Genet 2007;39(6):721-3.
28. Willcocks LC, Lyons PA, Clatworthy MR, et al. Copy number of FCGR3B, which is associated with systemic lupus erythematosus, correlates with protein expression and immune complex uptake. J Exp Med 2008;205(7):1573-82.

29. Tsao BP, Wu H. The genetics of human lupus. In: Wallace DJ, Hahn BH, editors. Dubois' lupus erythematosus. Philadelphia: Lippincott Williams & Wilkins; 2007. p. 54-81.
30. Freedman BI, Spray BJ, Heise ER, Espeland MA, Canzanello VJ. A race-controlled human leukocyte antigen frequency analysis in lupus nephritis. The South-Eastern Organ Procurement Foundation. Am J Kidney Dis 1993;21(4):378-82.
31. Harley JB, Sestak AL, Willis LG, Fu SM, Hansen JA, Reichlin M. A model for disease heterogeneity in systemic lupus erythematosus. Relationships between histocompatibility antigens, autoantibodies, and lymphopenia or renal disease. Arthritis Rheum 1989;32(7):826-36.
32. Hughes T, Kim-Howard X, Kelly JA, et al. Fine-mapping and transethnic genotyping establish IL2/IL21 genetic association with lupus and localize this genetic effect to IL21. Arthritis Rheum 2011;63(6):1689-97.
33. Jacob CO, Reiff A, Armstrong DL, et al. Identification of novel susceptibility genes in childhood-onset systemic lupus erythematosus using a uniquely designed candidate gene pathway platform. Arthritis Rheum 2007;56(12):4164-73.
34. Tang Y, Luo X, Cui H, et al. MicroRNA-146A contributes to abnormal activation of the type I interferon pathway in human lupus by targeting the key signaling proteins. Arthritis Rheum 2009;60(4):1065-75.
35. Sigurdsson S, Nordmark G, Goring HH, et al. Polymorphisms in the tyrosine kinase 2 and interferon regulatory factor 5 genes are associated with systemic lupus erythematosus. Am J Hum Genet 2005;76(3):528-37.
36. Kozyrev SV, Alarcon-Riquelme ME. The genetics and biology of Irf5-mediated signaling in lupus. Autoimmunity 2007;40(8):591-601.
37. Nath SK, Han S, Kim-Howard X, et al. A nonsynonymous functional variant in integrin-alpha(M) (encoded by ITGAM) is associated with systemic lupus erythematosus. Nat Genet 2008;40(2):152-4.
38. Han S, Kim-Howard X, Deshmukh H, et al. Evaluation of imputation-based association in and around the integrin-alpha-M (ITGAM) gene and replication of robust association between a non-synonymous functional variant within ITGAM and systemic lupus erythematosus (SLE). Hum Mol Genet 2009;18(6):1171-80.
39. Suarez-Gestal M, Calaza M, Endreffy E, et al. Replication of recently identified systemic lupus erythematosus genetic associations: a case-control study. Arthritis Res Ther 2009;11(3):R69.
40. Sawalha AH, Webb R, Han S, et al. Common variants within MECP2 confer risk of systemic lupus erythematosus. PLoS One 2008;3(3):e1727.
41. Webb R, Wren JD, Jeffries M, et al. Variants within MECP2, a key transcription regulator, are associated with increased susceptibility to lupus and differential gene expression in patients with systemic lupus erythematosus. Arthritis Rheum 2009;60(4):1076-84.
42. Rioux JD, Goyette P, Vyse TJ, et al. Mapping of multiple susceptibility variants within the MHC region for 7 immune-mediated diseases. Proc Natl Acad Sci U S A 2009;106(44):18680-5.
43. Prokunina L, Castillejo-Lopez C, Oberg F, et al. A regulatory polymorphism in PDCD1 is associated with susceptibility to systemic lupus erythematosus in humans. Nat Genet 2002;32(4):666-9.
44. Johansson M, Arlestig L, Moller B, Rantapaa-Dahlqvist S. Association of a PDCD1 polymorphism with renal manifestations in systemic lupus erythematosus. Arthritis Rheum 2005;52(6):1665-9.

45. Nielsen C, Laustrup H, Voss A, Junker P, Husby S, Lillevang ST. A putative regulatory polymorphism in PD-1 is associated with nephropathy in a population-based cohort of systemic lupus erythematosus patients. Lupus 2004;13(7):510-6.
46. Prokunina L, Gunnarsson I, Sturfelt G, et al. The systemic lupus erythematosus-associated PDCD1 polymorphism PD1.3A in lupus nephritis. Arthritis Rheum 2004;50(1):327-8.
47. Lee YH, Woo JH, Choi SJ, Ji JD, Song GG. Association of programmed cell death 1 polymorphisms and systemic lupus erythematosus: a meta-analysis. Lupus 2009;18(1):9-15.
48. Tan W, Sunahori K, Zhao J, et al. Association of PPP2CA polymorphisms with SLE susceptibility in multiple ethnic groups. Arthritis Rheum 2011.
49. Kyogoku C, Langefeld CD, Ortmann WA, et al. Genetic association of the R620W polymorphism of protein tyrosine phosphatase PTPN22 with human SLE. Am J Hum Genet 2004;75(3):504-7.
50. Wu H, Cantor RM, Graham DS, et al. Association analysis of the R620W polymorphism of protein tyrosine phosphatase PTPN22 in systemic lupus erythematosus families: increased T allele frequency in systemic lupus erythematosus patients with autoimmune thyroid disease. Arthritis Rheum 2005;52(8):2396-402.
51. Fernando MM, Stevens CR, Sabeti PC, et al. Identification of two independent risk factors for lupus within the MHC in United Kingdom families. PLoS Genet 2007;3(11):e192.
52. Moser KL, Kelly JA, Lessard CJ, Harley JB. Recent insights into the genetic basis of systemic lupus erythematosus. Genes Immun 2009;10(5):373-9.
53. Remmers EF, Plenge RM, Lee AT, et al. STAT4 and the risk of rheumatoid arthritis and systemic lupus erythematosus. N Engl J Med 2007;357(10):977-86.
54. Taylor KE, Remmers EF, Lee AT, et al. Specificity of the STAT4 genetic association for severe disease manifestations of systemic lupus erythematosus. PLoS Genet 2008;4(5):e1000084.
55. Ramos PS, Langefeld CD, Bera LA, Gaffney PM, Noble JA, Moser KL. Variation in the ATP-binding cassette transporter 2 gene is a separate risk factor for systemic lupus erythematosus within the MHC. Genes Immun 2009;10(4):350-5.
56. Cunninghame Graham DS, Graham RR, Manku H, et al. Polymorphism at the TNF superfamily gene TNFSF4 confers susceptibility to systemic lupus erythematosus. Nat Genet 2008;40(1):83-9.
57. Delgado-Vega AM, Abelson AK, Sanchez E, et al. Replication of the TNFSF4 (OX40L) promoter region association with systemic lupus erythematosus. Genes Immun 2009;10(3):248-53.
58. Lee-Kirsch MA, Gong M, Chowdhury D, et al. Mutations in the gene encoding the 3'-5' DNA exonuclease TREX1 are associated with systemic lupus erythematosus. Nat Genet 2007;39(9):1065-7.
