# Supplementary material for: A diverse array of genetic factors contribute to the pathogenesis of Systemic Lupus Erythematosus
Source: Orphanet J Rare Dis. 2013 Jan 7;8:2. doi: 10.1186/1750-1172-8-2 (PMC3551738; doi:10.1186/1750-1172-8-2)
Supplement: Additional file 2 — Table S2. Top 30 gene ontology functional annotations for SLE candidate genes [84,85]. [file 1750-1172-8-2-S2.pdf]

Supplementary data file S-2: Gene ontology annotation [1] of known genes for SLE, calculated using GOStat [2]

GO Term	Associated, Genes	No, of, genes (Total 67)	No, of, Term-Associated, Genes (Total 33972)	P-value
GO:0005515 Protein binding	trex1, traf6, cd44, actn4, kpna1, ppp2ca, scube1, notch4, ube2l3, tnfaip3, trim27, trim31, syk, jazf1, parvb, dnaja1, ptn, fcgr2a, ptpn22, rasgrp3, atg5, fcgr1a, ica1, tyk2, fcgr3a, rps6ka1, crp, fcgr2b, stat1, ets1, micb, tap2, stat4, pxk, lrrc18, fcgr1, bc4a, fcgr3b, pttg1, lyn, tnip1, c4b, mecp2, irak1, blk, tnfsf4, il21, spp1, itgam, il10, skiv2l	51	9005	$1.15x10^{-16}$
GO:0002376 Immune system process	fcgr1a, c1qa, fcgr3a, fcgr2b, c2, traf6, ets1, micb, tap2, hla-drb2, irf8, notch4, syk, fcgr1b, c4a, fcgr3b, bank1, lyn, hla-drb3, c4b, pdcd1, c1qb, fcgr2a, tnfsf4, il21, il10	26	1386	$1.59x10^{-16}$
GO:0006955 Immune response	fcgr1a, c1qa, fcgr3a, fcgr2b, c2, traf6, ets1, micb, tap2, hla-drb2, irf8, syk, fcgr1b, c4a, fcgr3b, hla-drb3, c4b, pdcd1, c1qb, fcgr2a, tnfsf4, il10	22	1189	$1.52x10^{-13}$
GO:0019865 Immunoglobin binding	fcgr1a, fcgr2a, fcgr3b, fcgr3a, fcgr2b, fcgr1b	6	16	$9.05x10^{-11}$
GO:0032403 Protein complex binding	trex1, fcgr1a, fcgr3b, fcgr3a, fcgr2b, actn4, fcgr2a, syk, fcgr1b	9	101	$9.17x10^{-11}$
GO:0019864 Ig G binding	fcgr1a, fcgr2a, fcgr3b, fcgr3a, fcgr2b	5	8	$2.34x10^{-10}$
GO:0051239 Regulator of multicellular organismal process	c4a, c1qa, c2, c4b, traf6, c1qb, pxk, il21, spp1, syk, il10	11	277	$1.04x10^{-09}$
GO:0048518	c4a, c1qa, lyn, c4b, c2, traf6, ets1, c1qb, actn4, ppp2ca, ptn, irak1,	17	1062	$2.18x10^{-09}$

Positive regulation of biological process	notch4, tnfsf4, il21, syk, il10			
GO:0002252<br>Immune effector process	c4a, c1qa, c2, c4b, traf6, c1qb, syk, il10	8	110	$5.66x10^{-09}$
GO:0065007<br>Biological regulation	c1qa, c2, ikzf1, traf6, actn4, kpna1, ppp2ca, irf8, scube1, notch4, ube2l3, irf5, tnfaip3, trim27, sykj, azf1, prdm1, c1qb, serpind1, ptn, rasgrp3, ets1, stat1, stat4, pxk, c4a, lyn, tnip1, c4b, mecp2, irak1, tnfsf4, il21, spp1, il10	35	6731	$7.29x10^{-09}$
GO:0050789<br>Regulation of biological process	c1qa, c2, ikzf1, traf6, stat1, ets1, stat4, actn4, kpna1, ppp2ca, notch4, irf8, pxk, ube2l3, irf5, tnfaip3, trim27, syk, jazf1, c4a, lyn, prdm1, tnip1c4b, mecp2, c1qb, irak1, ptn, tnfsf4, il21, rasgrp3, spp1, il10	33	6140	$8.18x10^{-09}$
GO:0002253<br>Activation of immune response	c4a, c1qa, c2, c4b, syk, traf6, c1qb	7	71	$8.28x10^{-09}$
GO:0007243<br>Protein kinase cascade	lyn, traf6, stat1, ppp2ca, stat4, blk, irak1, tnfaip3, rasgrp3, syk, il10	11	376	$1.39x10^{-08}$
GO:0002443<br>Leukocyte mediated immunity	c1qa, c2, c4b, syk, traf6, c1qb, il10	7	78	$1.39x10^{-08}$
GO:0050778<br>Positive regulation of immune response	c4a, c1qa, c2, c4b, syk, traf6, c1qb	7	85	$2.41x10^{-08}$
GO:0002684<br>Positive regulation of immune system process	c4a, c1qa, c2, c4b, syk, traf6, c1qb	7	86	$2.45x10^{-08}$
GO:0050776<br>Regulation of immune response	c4a, c1qa, c2, c4b, syk, traf6, c1qb	7	98	$5.83x10^{-08}$

GO:0002682<br>Regulation of immune system process	c4a, c1qa, c2, c4b, syk, traf6, c1qb	7	100	$6.35x10^{-08}$
GO:0051240<br>Positive regulation of multicellular organismal process	c4a, c1qa, c2, c4b, syk, traf6, c1qb	7	105	$8.49x10^{-08}$
GO:0002449<br>Lymphocyte mediated immunity	c1qa, c2, c4b, traf6, c1qb, il10	6	72	$3.12x10^{-07}$
GO:0005886<br>Plasma membrane	fcgr1a, or2h2, fcgr3a, fcgr2b, cd44, micb, ppp2ca, hla-drb2, scube1, notch4, pxk, trim27syk, fcgr1b, parvb, fcgr3b, lyn, hla-drb3, irak1, fcgr2a, tnfsf4, rasgrp3, itgam	23	3816	$3.12x10^{-07}$
GO:0032501<br>Multicellular organismal process	or2h2, c1qa, c2, ikzf1, traf6, ets1, ppp2ca, irf8, scube1, notch4, pxk, syk, c4a, lyn, c4b, pdcd1c1qb, serpind1, ptn, ptpn22, il21, spp1, il10	23	3822	$3.13x10^{-07}$
GO:0006959<br>Humoral immune response	c4a, c1qa, c2, c4b, pdcd1, c1qb	6	75	$3.61x10^{-07}$
GO:0002460<br>adaptive immune response based on somatic recombination of immune receptors built from immunoglobulin superfamily domains	c1qa, c2, c4b, traf6, c1qb, il10	6	76	$3.61x10^{-07}$
GO:0002250<br>Adaptive immune response	c1qa, c2, c4b, traf6, c1qb, il10	6	76	$3.61x10^{-07}$
GO:0002526<br>Acute Inflammatory response	c4a, c1qa, crp, c2, c4b, c1qb	6	79	$4.39x10^{-07}$

GO:0006956<br>Complement Activation	c4a, c1qa, c2, c4b, c1qb	5	39	$4.91x10^{-07}$
GO:0002541<br>Inactivation of plasma proteins during acute inflammatory response	c4a, c1qa, c2, c4b, c1qb	5	39	$4.91x10^{-07}$
GO:0006952<br>Defense response	c4a, or2h2, c1qa, crp, tnip1, c2, c4b, c1qb, micb, scube1, il10	11	584	$6.43x10^{-07}$
GO:0016064<br>Immunoglobin mediated immune response	c1qa, c2, c4b, c1qb, il10	5	51	$1.84x10^{-06}$

References:

1. Camon E, Magrane M, Barrell D, et al. The Gene Ontology Annotation (GOA) Database: sharing knowledge in Uniprot with Gene Ontology. Nucleic Acids Res 2004;32(Database issue):D262-6.
2. Beissbarth T, Speed TP. GOstat: find statistically overrepresented Gene Ontologies within a group of genes. Bioinformatics 2004;20(9):1464-5.
