# Supplementary material for: A diverse array of genetic factors contribute to the pathogenesis of Systemic Lupus Erythematosus
Source: Orphanet J Rare Dis. 2013 Jan 7;8:2. doi: 10.1186/1750-1172-8-2 (PMC3551738; doi:10.1186/1750-1172-8-2)
Supplement: Additional file 3 — Diagram S3. Relationships between top gene ontology functional terms for SLE candidate genes. [file 1750-1172-8-2-S3.pdf]

<u>Supplementary Data File S-3.</u> Mapping of Gene Ontology terms[1] over-represented in genes already known to be associated with SLE (from www.geneontology.org).

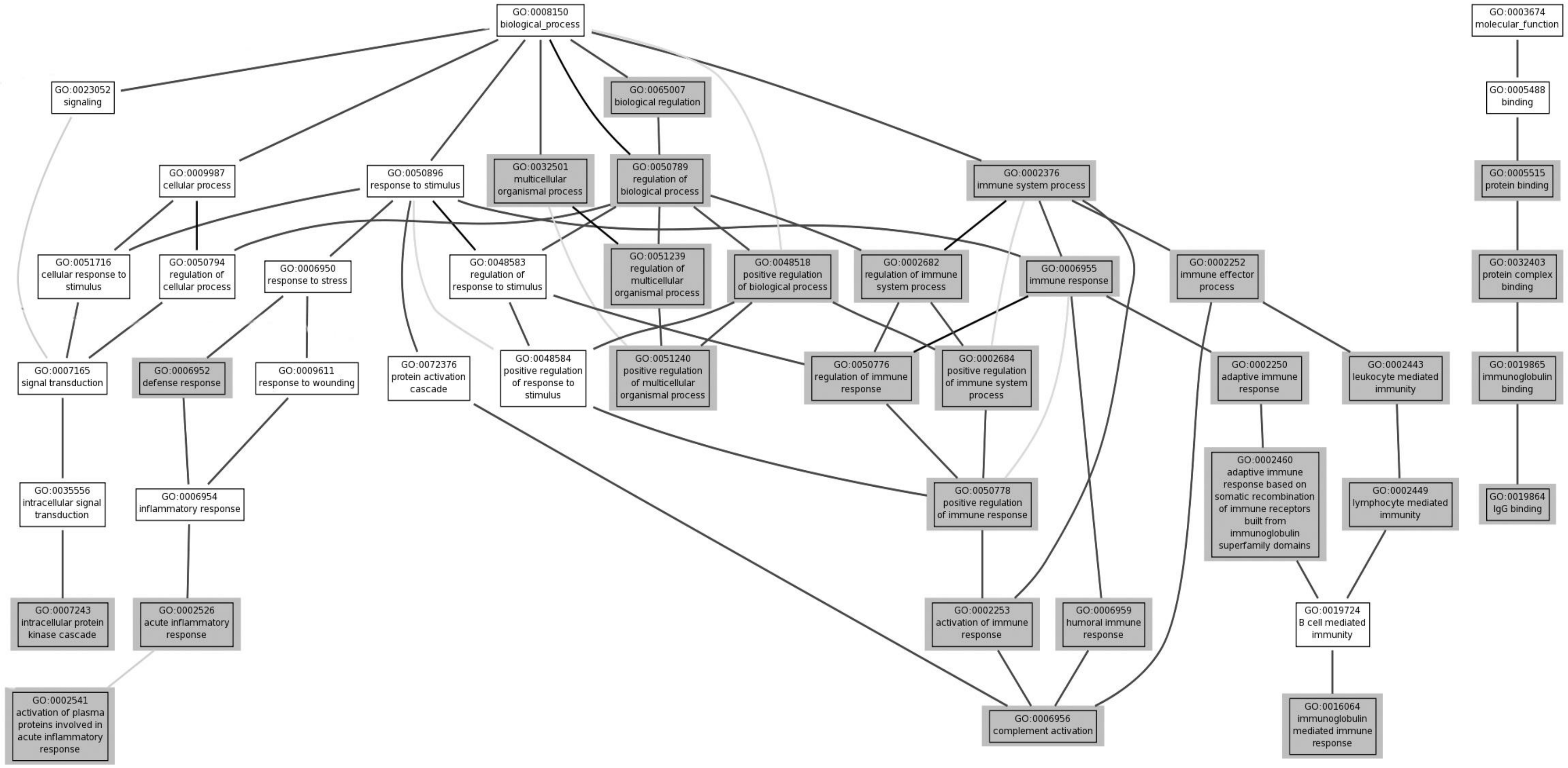

<u>References:</u>

1. Camon E, Magrane M, Barrell D, et al. The Gene Ontology Annotation (GOA) Database: sharing knowledge in Uniprot with Gene Ontology. Nucleic Acids Res 2004;32(Database issue):D262-6.
