# Supplementary material for: A diverse array of genetic factors contribute to the pathogenesis of Systemic Lupus Erythematosus
Source: Orphanet J Rare Dis. 2013 Jan 7;8:2. doi: 10.1186/1750-1172-8-2 (PMC3551738; doi:10.1186/1750-1172-8-2)
Supplement: Additional file 4 — Table S4. microRNA molecules implicated in SLE [86]. [file 1750-1172-8-2-S4.pdf]

Supplementary data file S-4: **MicroRNAs that have been implicated in SLE.**

**miRNA**	**Regulates:**	**Expression in SLE; hypothesis of action:**	**Ref.**
miR-112		Downregulated	[1]
miR-1224-3p	GPDH, PMVK, BSG	Downregulated	[2]
miR-1224-3P		Downregulated	[2]
miR-125a	KLF13	Downregulated. Indirectly regulates RANTES via KLF13 (inflammation)	[3]
miR-126		Upregulated. Regulates DNA methylation by reduction in DMNT1 levels.	[4]
miR-141		Downregulated	[1]
miR-142-3p		Upregulated	[1]
miR-146a		Downregulated. Assayed in PBMCs.	[5]
miR-146a		Serum levels downregulated. Urinary levels upregulated.	[6]
miR-146a	IRF-5, STAT-1, IRAK1, TRAF6	Downregulated. Directly represses transactivation of IFNG1; suppress inflammatory cytokine production.	[7]
miR-146a		Promoter mutation of miRNA molecule results in reduced binding of Ets1 and reduced expression of miR-146a, with concomitant increase in IFN1 activation	[8]
miR-148a	DNMT1, DNMT3B	Upregulated. Directly down regulates DNMT1 leading to hypomethylation (epigenetic effect)	[9]
miR-155		Upregulated in regulatory T cells	[10]
miR-155		Serum levels downregulated.	[6]
miR-17-5p		Downregulated	[1]
miR-181		Downregulated. Is an important modulator of B and T cell differentiation, maturation and function – downregulated in paediatric SLE	[11]
miR-184		Downregulated	[1]
miR-189		Upregulated	[1]
miR-196a		Downregulated	[1]
miR-198		Upregulated	[1]
miR-21	RASGRP1	Upregulated. Indirectly downregulates DNMT1 expression via RASGRP1, causing hypomethylation (epigenetic effect)	[9]
miR-21	PDCD4	Upregulated. Decreases expression of PDCD4, a selective protein translation inhibitor that affects T cell response.	[12]
miR-21		Upregulated	[1]
miR-298		Upregulated	[1]
miR-299-3p		Upregulated	[1]

miR-342		Upregulated	[1]
miR-371-5p	IL-32, IFIT3, IFIT2, FGR, IRF5, CD40, PTTG1	Upregulated	[2]
miR-383		Downregulated	[1]
miR-409-3p		Downregulated	[1]
miR-423-5p	SLC2A4, VGF, SOX12	Upregulated	[2]
miR-61		Upregulated	[1]
miR-638	CD79B, LY6E, ZNF330	Upregulated.	[2]
miR-663	IL-32, IFI35, CENTA1, LY6E, ZNF330	Upregulated.	[13]
miR-663		Upregulated	[2]
miR-78		Upregulated	[1]

References:

1. Dai Y, Huang YS, Tang M, et al. Microarray analysis of microRNA expression in peripheral blood cells of systemic lupus erythematosus patients. Lupus 2007;16(12):939-46.
2. Te JL, Dozmorov IM, Guthridge JM, et al. Identification of unique microRNA signature associated with lupus nephritis. PLoS One 2010;5(5):e10344.
3. Zhao X, Tang Y, Qu B, et al. MicroRNA-125a contributes to elevated inflammatory chemokine RANTES levels via targeting KLF13 in systemic lupus erythematosus. Arthritis Rheum 2010;62(11):3425-35.
4. Zhao S, Wang Y, Liang Y, et al. MicroRNA-126 regulates DNA methylation in CD4+ T cells and contributes to systemic lupus erythematosus by targeting DNA methyltransferase 1. Arthritis Rheum 2011;63(5):1376-86.
5. Hai-yan W, Yang L, Mei-hong C, Hui Z. Expression of MicroRNA-146a in peripheral blood mononuclear cells in patients with systemic lupus Erythematosus. Zhongguo Yi Xue Ke Xue Yuan Xue Bao 2011;33(2):185-8.
6. Wang G, Tam LS, Li EK, et al. Serum and urinary cell-free MiR-146a and MiR-155 in patients with systemic lupus erythematosus. J Rheumatol 2010;37(12):2516-22.
7. Tang Y, Luo X, Cui H, et al. MicroRNA-146A contributes to abnormal activation of the type I interferon pathway in human lupus by targeting the key signaling proteins. Arthritis Rheum 2009;60(4):1065-75.
8. Luo X, Yang W, Ye DQ, et al. A Functional Variant in MicroRNA-146a Promoter Modulates Its Expression and Confers Disease Risk for Systemic Lupus Erythematosus. PLoS Genet 2011;7(6):e1002128.
9. Pan W, Zhu S, Yuan M, et al. MicroRNA-21 and microRNA-148a contribute to DNA hypomethylation in lupus CD4+ T cells by directly and indirectly targeting DNA methyltransferase 1. J Immunol 2010;184(12):6773-81.
10. Divekar AA, Dubey S, Gangalum PR, Singh RR. Dicer insufficiency and microRNA-155 overexpression in lupus regulatory T cells: an apparent paradox in the setting of an inflammatory milieu. J Immunol 2011;186(2):924-30.
11. Lashine YA, Seoudi AM, Salah S, Abdelaziz AI. Expression signature of microRNA-181-a reveals its crucial role in the pathogenesis of paediatric systemic lupus erythematosus. Clin Exp Rheumatol 2011;29(2):351-7.

12. Stagakis E, Bertsias G, Verginis P, et al. Identification of novel microRNA signatures linked to human lupus disease activity and pathogenesis: miR-21 regulates aberrant T cell responses through regulation of PDCD4 expression. Ann Rheum Dis 2011.
13. Dai Y, Sui W, Lan H, Yan Q, Huang H, Huang Y. Comprehensive analysis of microRNA expression patterns in renal biopsies of lupus nephritis patients. Rheumatol Int 2009;29(7):749-54.
