# Supplementary material for: A diverse array of genetic factors contribute to the pathogenesis of Systemic Lupus Erythematosus
Source: Orphanet J Rare Dis. 2013 Jan 7;8:2. doi: 10.1186/1750-1172-8-2 (PMC3551738; doi:10.1186/1750-1172-8-2)
Supplement: Additional file 5 — Table S5. Mouse models for SLE. [file 1750-1172-8-2-S5.pdf]

Supplementary data file S-5: Mouse models of SLE as defined in the Mouse Genome Informatics Database (http://www.informatics.jax.org/) [1].

**Human Disease**	Term: **Systemic Lupus Erythematosus; SLE**<br>**OMIM ID:** 152700
**Associated Genes**	Orthologous mouse and human markers where mutations in one or both species have been associated with phenotypes characteristic of this disease.

Mouse Gene	Human Gene	Characteristics of this human disease are associated with mutations in...
**Dnase1**<br>**Fcgr2b**<br>**Ptpn22**	**DNASE1**<br>**FCGR2B**<br>**PTPN22**	**...both mouse and human orthologous genes.**
**Lyz2**<br>**C1qa**<br>**C4b**<br>**Ccr2**<br>**Cd19**<br>**Cd4**<br>**Cdkn1a**<br>**Csf2**	none identified<br>C1QA<br>C4B<br>CCR2<br>CD19<br>CD4<br>CDKN1A<br>CSF2	**...the mouse gene.**<br>*OMIM data currently do not associate this disease with the orthologous human gene.*

**Def6**	DEF6
**Ep300**	EP300
**Fas**	FAS
**Gadd45a**	GADD45A
**Ikzf3**	IKZF3
**Il3**	IL3
**Junb**	JUNB
**Kit**	KIT
**Lyn**	LYN
**Man2a1**	MAN2A1
**Mta2**	MTA2
**Pparg**	PPARG
**Psen1**	PSEN1
**Psen2**	PSEN2
**Ptprc**	PTPRC
**Rasgrp1**	RASGRP1
**Rassf5**	RASSF5
**Rc3h1**	RC3H1
**Rxra**	RXRA
**Tnfrsf13b**	TNFRSF13B
**Tnfrsf9**	TNFRSF9
**Trove2**	TROVE2

	Bank1	**BANK1**	**...the human gene.** *MGI data currently do not associate this disease with mutations in the orthologous mouse gene.*
	C4a	**C4A**	
	Fasl	**FASLG**	
	Fcgr4	**FCGR3A**	
	Itgax	**ITGAX**	
	Pdcd1	**PDCD1**	
	none identified	**SLEB1**	
	none identified	**SLEB3**	
	none identified	**SLEB4**	
	none identified	**SLEB5**	
	none identified	**SLEH1**	
	Stat4	**STAT4**	
	Trex1	**TREX1**	
**Transgenes and other mutation types**	Igh	Characteristics of this human disease are associated with transgenes and other mutation types in mouse.	

Genotype			Ref(s)
**Allelic Composition**	**Note**	**Genetic Background**	
**Models with phenotypic similarity to human disease where etiologies involve orthologs.[1]**			
Dnase1^{tm1Tmo}/Dnase1^{tm1Tmo}		involves: 129P2/OlaHsd * C57BL/6	J:62549
Fcgr2b^{tm1Ttk}/Fcgr2b^{tm1Ttk}		B6.129S4-Fcgr2b^{tm1Ttk}	J:64152
Ptpn22^{tm2Achn}/Ptpn22^{tm2Achn} Ptprc^{tm1Weis}/Ptprc^{tm1Weis}		B6.Cg-Ptprc^{tm1Weis} Ptpn22^{tm2Achn}	J:147129
**Models with phenotypic similarity to human disease where etiologies are distinct.[2]**			
C1qa^{tm1Mjw}/C1qa^{tm1Mjw}		involves: 129P2/Ola * 129S/SvEv * C57BL/6	J:47315
C4b^{tm1Crr}/C4b^{tm1Crr}		involves: 129S4/SvJae * C57BL/6J	J:111811
Ccr2^{tm1Blck}/Ccr2^{tm1Blck} Fas^{lpr}/Fas^{lpr}		MRL.Cg-Ccr2^{tm1Blck} Fas^{lpr}	J:113343
Cd19$^{tm1(cre)Cgn}$/Cd19^{+} Ep300^{tm2Reck}/Ep300^{+}	5	involves: 129/Sv * 129P2/OlaHsd * C57BL/6	J:147840
Cd4^{tm1Mak}/Cd4^{tm1Mak} Igh-J$^{tm2(3H9-VDJ*)Mwg}$/Igh-J^{+}		B6.Cg-Cd4^{tm1Mak} Igh-J$^{tm2(3H9-VDJ*)Mwg}$	J:142389
Cdkn1a^{tm1Led}/Cdkn1a^{tm1Led} Gadd45a^{tm1Ajf}/Gadd45a^{tm1Ajf}		either: (involves: 129P2/OlaHsd * 129S6/SvEvTac * C57BL/6) or (involves: 129S6/SvEvTac * 129X1/SvJ * C57BL/6)	J:76041
Cdkn1a^{tm1Led}/Cdkn1a^{tm1Led}		involves: 129S6/SvEvTac	J:76041
Csf2^{tm1Dran}/Csf2^{tm1Dran} Il3^{tm1Glli}/Il3^{tm1Glli}		B6.129S2-Csf2^{tm1Dran} Il3^{tm1Glli}	J:83086
Def6$^{Gt(OST307148)Lex}$/Def6$^{Gt(OST307148)Lex}$		involves: 129S5/SvEvBrd * C57BL/6	J:106469
Fas^{lpr}/Fas^{lpr} Tnfrsf9^{tm1Byk}/Tnfrsf9^{tm1Byk}		MRL.Cg-Tnfrsf9^{tm1Byk} Fas^{lpr}	J:120559
Fas^{lpr}/Fas^{lpr} Igh-J$^{tm2(3H9-VDJ*)Mwg}$/Igh-J^{+}		MRL.Cg-Fas^{lpr} Igh-J$^{tm2(3H9-VDJ*)Mwg}$	J:131138
Fas^{lpr}/Fas^{lpr}		MRL/Mp-Fas^{lpr}	J:27634, J:28885,J:108760, J:131138
Gadd45a^{tm1Ajf}/Gadd45a^{tm1Ajf}		either: (involves: 129P2/OlaHsd * C57BL/6) or (involves: 129X1/SvJ * C57BL/6)	J:76041
Ikzf3^{tm1Kge}/Ikzf3^{tm1Kge}		involves: 129S4/SvJae * C57BL/6	J:50626, J:81805

$Junb^{tm3Wag}$/$Junb^{tm3Wag}$<br>Tg(KRT5-cre)1Tak/0	5	involves: 129/Sv * 129P2/OlaHsd * C3H * C57BL/6	J:155575
Kit^{W-sh}/Kit^{W-sh}<br>Lyn^{tm1Ard}/Lyn^{tm1Ard}		involves: 101 * 129P2/OlaHsd * C3H/HeH	J:161523
Lyn^{tm1Ard}/Lyn^{tm1Ard}		involves: 129P2/OlaHsd	J:161523
$Lyz2^{tm1(cre)Ifo}$/$Lyz2^{+}$<br>$Rxra^{tm1Krc}$/$Rxra^{tm1Krc}$	5	involves: 129P2/OlaHsd * 129S4/SvJae * C57BL/6	J:168799
$Lyz2^{tm1(cre)Ifo}$/$Lyz2^{+}$<br>$Pparg^{tm1.1Gonz}$/$Pparg^{tm1.1Gonz}$	5	involves: 129P2/OlaHsd * 129X1/SvJ * C57BL/6	J:168799
$Man2a1^{tm1Jxm}$/$Man2a1^{tm1Jxm}$		involves: 129S1/Sv * 129X1/SvJ * C57BL/6	J:74579
$Mta2^{tm1.1Yzha}$/$Mta2^{tm1.1Yzha}$		involves: 129S4/SvJae * C57BL/6 * FVB/N	J:137101
$Psen1^{tm1Bdes}$/$Psen1^{+}$<br>$Psen2^{tm1Bdes}$/$Psen2^{tm1Bdes}$		involves: 129P2/OlaHsd	J:91235
$Ptprc^{tm1Weis}$/$Ptprc^{tm1Weis}$		involves: 129X1/SvJ * C57BL/6	J:66501
$Ptprc^{tm1Weis}$/$Ptprc^{+}$		involves: 129X1/SvJ * C57BL/6	J:66501
$Rasgrp1^{lag}$/$Rasgrp1^{lag}$		involves: 129 * C57BL/6	J:85189
$Rassf5^{tm1Kina}$/$Rassf5^{tm1Kina}$		B6.Cg-$Rassf5^{tm1Kina}$	J:168749
$Rc3h1^{san}$/$Rc3h1^{san}$		either: C57BL/6-$Rc3h1^{san}$ or (involves: C57BL/6 * CBA/Ca)	J:98938
$Tnfrsf13b^{tm1Vmd}$/$Tnfrsf13b^{tm1Vmd}$		involves: C57BL/6	J:82331
$Trove2^{tm1Woln}$/$Trove2^{tm1Woln}$		involves: 129S1/Sv * C57BL/6	J:84627
$Trove2^{tm1Woln}$/$Trove2^{tm1Woln}$		B6.129S1-$Trove2^{tm1Woln}$	J:84627
$Trove2^{tm1Woln}$/$Trove2^{+}$		involves: 129S1/Sv * C57BL/6	J:84627
**Models involving transgenes or other mutation types.[3]**			
$Cd4^{tm1Mak}$/$Cd4^{tm1Mak}$<br>$Igh\text{-}J^{tm2(3H9\text{-}VDJ*)Mwg}$/$Igh\text{-}J^{+}$		B6.Cg-$Cd4^{tm1Mak}$ $Igh\text{-}J^{tm2(3H9\text{-}VDJ*)Mwg}$	J:142389
Fas^{lpr}/Fas^{lpr}<br>$Igh\text{-}J^{tm2(3H9\text{-}VDJ*)Mwg}$/$Igh\text{-}J^{+}$		MRL.Cg-Fas^{lpr} $Igh\text{-}J^{tm2(3H9\text{-}VDJ*)Mwg}$	J:131138
$Igh\text{-}J^{tm2(3H9\text{-}VDJ*)Mwg}$/$Igh\text{-}J^{+}$		B6.129P2-$Igh\text{-}J^{tm2(3H9\text{-}VDJ*)Mwg}$	J:142389
$Igh^{tm1(Igh564)Tik}$/$Igh^{tm1(Igh564)Tik}$<br>$Igk^{tm1(Igk564)Tik}$/$Igk^{tm1(Igk564)Tik}$		B6.129S4-$Igk^{tm1(Igk564)Tik}$ $Igh^{tm1(Igh564)Tik}$	J:113555
$Tcrb^{tm1Mom}$/$Tcrb^{tm1Mom}$<br>$Tcrd^{tm1Mom}$/$Tcrd^{tm1Mom}$		B6.129P2-$Tcrb^{tm1Mom}$ $Igh\text{-}J^{tm2(3H9\text{-}VDJ*)Mwg}$ $Tcrd^{tm1Mom}$	J:142389

Igh-J$^{tm2(3H9-VDJ*)Mwg}$/Igh-J^{+}			
Tg(ACTB-TNFRSF6B)754Jwu/?		B6.Cg-Tg(Actb-TNFRSF6B)754Jwu	J:148162
Tg(Igh-V-CD40lg)#Tsub/0		C57BL/6-Tg(Igh-V-CD40lg)#Tsub	J:128824
X/Yaa		BXSB	J:7308, J:108760
X/Yaa		BXSB/Mp	J:6235
X/Yaa		(NZW x BXSB)F1	J:7276
X/Yaa		(NZW x SB)F1	J:7276
X/Yaa		(NZB x BXSB)F1	J:6235
X/Yaa		BXSB/MpJScr	J:10973
**No similarity to the expected human disease phenotype was found.[4]**			
NOT Csf2^{tm1Dran}/Csf2^{tm1Dran} Ifng^{tm1Ts}/Ifng^{tm1Ts} Il3^{tm1Glli}/Il3^{tm1Glli}		involves: 129S2/SvPas * 129S7/SvEvBrd * C57BL/6	J:83086

[1]Human genes are associated with this disease. Mouse model genotypes include mutations in the orthologs of these human genes.
[2]Human genes are associated with this disease. Mouse model genotypes do not include mutations in the orthologs of these human genes.
[3]Models involving transgenes or other mutation types may also appear in other sections of the table.
[4]One or more human genes are associated with this human disease. The mouse genotype may involve mutations to orthologs of one or more of those genes, but the phenotype did not resemble the disease.
[5]Conditionally targeted allele(s)

References:

1. Blake JA, Bult CJ, Kadin JA, Richardson JE, Eppig JT. The Mouse Genome Database (MGD): premier model organism resource for mammalian genomics and genetics. Nucleic Acids Res 2011;39(Database issue):D842-8.
